# Analysis of mutation spectrum of common deafness-causing genes in Hakka newborns in southern China by semiconductor sequencing

**DOI:** 10.1097/MD.0000000000012285

**Published:** 2018-09-21

**Authors:** Pingsen Zhao, Lifang Lin, Liubing Lan

**Affiliations:** a Clinical Core Laboratory; b Center for Precision Medicine, Meizhou People's Hospital (Huangtang Hospital), Meizhou Hospital Affiliated to Sun Yat-sen University; c Guangdong Provincial Engineering and Technology Research Center for Molecular Diagnostics of Cardiovascular Diseases; d Meizhou Municipal Engineering and Technology Research Center for Molecular Diagnostics of Cardiovascular Diseases; e Meizhou Municipal Engineering and Technology Research Center for Molecular Diagnostics of Major Genetic Disorders; f Prenatal Diagnosis Center, Meizhou People's Hospital (Huangtang Hospital), Meizhou Hospital Affiliated to Sun Yat-sen University, Meizhou, P. R. China.

**Keywords:** deafness-causing genes, *GJB*, *GJB3*, Hakka population, mitochondrial genes, semiconductor sequencing, *SLC26A4*

## Abstract

Hearing loss is a common neurosensory disorder, approximately half of the cases are caused by genetic factors, and approximately 70% of hereditary hearing impairments are nonsyndromic hearing loss (NSHL). The mutations of *GJB2* (gap junction beta-2 protein)*, GJB3* (gap junction beta-3 protein), *SLC26A4* (solute carrier family 26 member 4), and *MT-RNR1* (mitochondrially encoded 12S RNA) are the most common inherited causes of NSHL. Because of different genetic backgrounds, the mutation spectrum of these common deafness-causing genes varies among different regions in China. Because no data are known on these mutations among the Hakka population of Southern China, we aim to investigate the mutation spectrum to add these to neonatal screening and genetic counseling. A total of 1252 blood samples from newborns have been detected by semiconductor sequencing for 100 mutations loci of 18 deafness-causing genes. Of the participants, 95 subjects carried deafness-causing genes mutations with the carrier rate of 7.59%. The mutation frequencies of *GJB2*, *SLC26A4*, *GJB3*, and mitochondrial genes were 3.04%, 3.51%, 0.16%, and 0.88%, respectively. We followed up subjects with single-gene homozygous or compound heterozygous mutations. Our study firstly analyzed deafness-causing genes mutation spectrum in Hakka population, providing evidence for future neonatal screening and genetic counseling in this area.

## 1 Introduction

Hearing loss is one of the most common neurosensory disorders, affecting approximately 1 to 3 newborns in every 1000 live births.^[1–3]^ In China, there are approximately 800,000 children younger than 7 years who are hearing impaired, and this number continues to grow, with an increase of more than 30,000 deaf children every year.^[3,4]^ According to whether other organ systems are abnormal, sensorineural hearing loss can be classified into nonsyndromic hearing loss (NSHL) and syndrome-induced hearing loss. If infants with profound hearing loss are not detected and treated within the first year of life, they may experience permanent hearing impairment with major and irreversible defects in linguistic and cognitive development. However, this situation can be improved if it is detected and intervention started before 6 months of age (cochlear implants can help patients with severe hearing loss to recover their hearing ability, implanting an electronic medical device that sends sound signals to the brain to replace the damaged inner ear.).

Previous studies have confirmed that hearing loss can be congenital or caused by environmental factors, such as infection, trauma, or ototoxic drugs.^[5,6]^ However, hearing loss is etiologically uneven. Some studies have shown that at least two-thirds of the cases of childhood-onset hearing loss have a genetic cause, and approximately 70% of hereditary hearing impairments are NSHL.^[7–9]^ NSHL can be inherited by autosomal recessive, autosomal dominant, X-linked trait, or mitochondrial deafness.^[10–12]^ At present, previous genetic screening studies have shown that a few genes mutations are known to cause hereditary hearing loss or deafness, such as *GJB2* (gap junction beta-2 protein; OMIM: 121011), *GJB3* (gap junction beta-2 protein; OMIM: 603324), *SLC26A4* (solute carrier family 26 member 4; OMIM: 605646), and the mitochondrial gene *MT-RNR1* (mitochondrially encoded 12S RNA; OMIM: 561000).^[9,13–24]^ Knowledge of the gene mutation can help to identify hearing impairment at birth, and the educational programs for auditory stimulation and sufficient language exposure in early childhood can begin immediately. Furthermore, it can also provide warning to avoid taking certain types of aminoglycosides antibiotics, such as streptomycin, gentamicin, and tobramycin, which are known to cause deafness in children carrying certain mitochondrial gene mutations.

China is the most populous country in the world, consisting of 56 nationalities. Due to geographical separation, Chinese people from different regions may have different genetic backgrounds.^[25,26]^ The Hakka population is a Han Chinese that mainly living in southern China with unique culture.^[26]^ The city Meizhou with the most Hakka population in Guangdong Province locates in the south part of China. Because of its remote location, Meizhou city is a relatively conservative area with a less migration of population. Hearing loss in infancy is a common sensory disorder, of which about is hereditary, caused by known mutations such as *GJB2, SLC26A4, GJB3*, and mitochondrial genes. Although some genetic studies have been performed on Chinese patients with deafness,^[8,9,27,28]^ the large-scale deafness population and racial differences require regional and individual genetic analysis; these data cannot simply be inferred from the conclusions of other groups. For example, 1 study with a comprehensive investigation of the molecular etiology of nonsyndromic deafness in 2 typical areas from northern and southern China (Chifeng City in Inner Mongolia and Nantong City in Jiangsu Province), *GJB2* gene mutations account for approximately 18.31% of patients with hearing impairment, *SLC26A4* gene mutations account for approximately 13.73%, and the mitochondrial m.1555A > G mutation accounts for 1.76%.^[8]^ However, common molecular etiologies are rare in the Tibetan Chinese deaf population.^[27]^ The prevalence of mutations varies among different regions in China. We aim to explore the prevalence of these mutations among the Hakka population in southern China, which might be helpful to neonatal screening and genetic counseling.

## 2 Materials and methods

### 2.1 Participants

**Figure 1:**
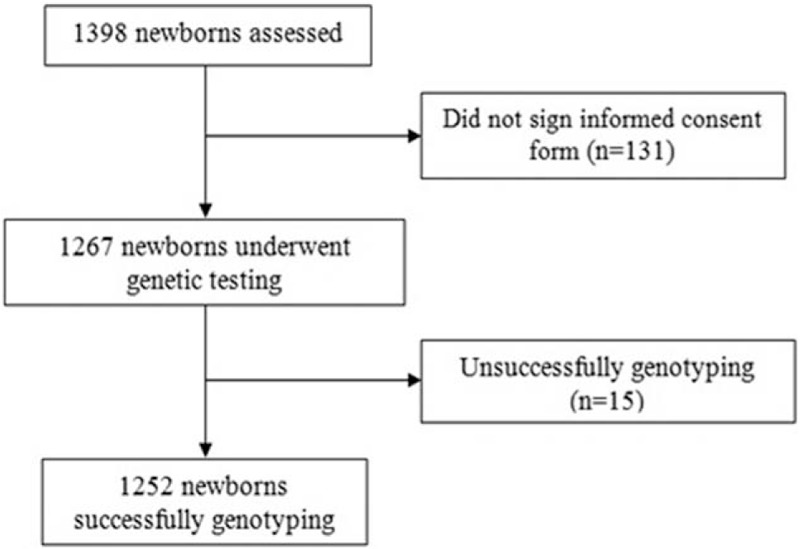
Flow chart of participant inclusion and exclusion.

This retrospective clinical study included 1252 newborns who born in Meizhou People's Hospital (Huangtang Hospital), Meizhou Hospital Affiliated to Sun Yat-sen University between May 2016 and January 2018. The inclusion and exclusion criteria are shown in Figure 1. All the blood samples from participants have been detected by semiconductor sequencing. Before blood sampling, informed consent was obtained from all participants’ parents. The study was approved by the Committee of Ethics and Research of the Meizhou People's Hospital, Meizhou Hospital Affiliated to Sun Yat-sen University for experiments involving humans. Before participants recruited for the study, their guardians signed a written informed consent form according to the ethical guidelines of the Helsinki Declaration.

### 2.2 DNA extraction and detection of deafness-causing genes mutations

Peripheral blood samples were collected from the study participants and stored in 2-mL evacuated vacuum tubes containing ethylenediaminetetraacetic, or heel blood was collected to the blood spot card. These blood samples are stored at 4°C for not more than 3 days before testing. QIAamp DNA Blood Mini Kit (Qiagen, Germany) was used to extract genomic DNA from each blood sample following the manufacturer's instructions, and NanoDrop 2000 Spectrophotometer (Thermo Fisher Scientific, Waltham, MA) was used to evaluate the quantity and quality of extracted DNA. DNA from peripheral blood sample was used for library construction according to the Ion Plus Fragment Library Kit (Life Technologies, Carlsbad, CA), and semiconductor sequencing was performed using an Ion Proton instrument according to the manufacturer's instructions (Life Technologies). High-throughput sequencing technology and bioinformatics analysis methods were used to detect the presence of deafness-causing gene mutation in subjects. The detection contents include 18 deafness-causing genes, including *GJB2*, *SLC26A4*, *GJB3*, *MYO15A* (unconventional myosin-15), *TECTA* (tectorin alpha), *DIABLO* (Diablo IAP-binding mitochondrial protein), *COCH* (cochlin), *DSPP* (dentin sialophosphoprotein), *GPR98* (G-protein coupled receptor 98), *DFNA5* (deafness, autosomal dominant 5), *TMC1* (transmembrane channel like 1), *MT-CO1* (mitochondrially encoded cytochrome C oxidase I), *MT-RNR1* (mitochondrially encoded TRNA histidine), *MT-TH* [mitochondrially encoded TRNA serine 1 (UCN)], *MT-TS1* [mitochondrially encoded TRNA leucine 1 (UUA/G)], MT-TL1 [mitochondrially encoded TRNA leucine 1 (UUA/G)], *PRPS1* (phosphoribosyl pyrophosphate synthetase 1), *MYO7A* (unconventional myosin-VIIa), a total of 100 mutations loci, of these 91% (91/100) mutations loci are invariant, the other 9 mutations loci are updated based on pathogenicity.

## 3 Results

Among the 1252 participants enrolled in this study (669 men and 583 women), 95 subjects carried common deafness-causing genes mutations, total carrier rate was 7.59% (Table 1). A total of 38 participating subjects carried mutations in *GJB2*, and the carrier rate was 3.04% in the population. *SLC26A4* mutations were detected in 44 participants; the carrier rate was 3.51%. Two participants carried mutations in *GJB3*. Mitochondrial gene mutations carrier rates were *MT-RNR1* (0.64%), *MT-TL1* (0.08%), and *MT-CO1* (0.16%). Mutations of *GJB2* and *SLC26A4* are major ones (86.32% of total).

**Table 1:**
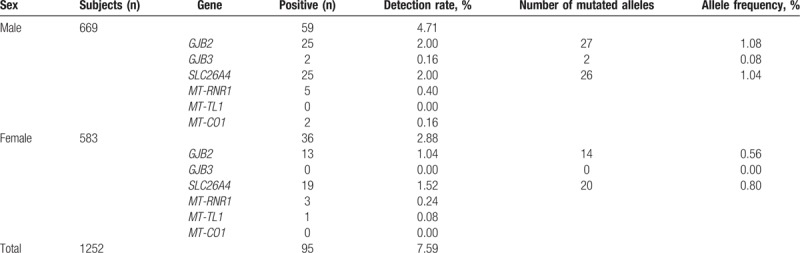
Deafness gene mutation carrier rates detected in 1252 Hakka newborns.

### 3.1 Mutations in *GJB2* gene

Four variants were identified in this cohort. They were 2 frameshift deletions (c.235delC, c.299_300delAT), 1 frameshift insertion (c.511_512insAACG) and 1 missense mutations (c.109G>A) (Table 2). All of the variants were pathological mutations, which have been determined in previous studies. The mutant alleles of *GJB2* accounted for 1.64% (41/2504) of the total alleles in all subjects (Table 2). Like most areas of China, the most common mutation allele of *GJB2* in Hakka area was c.235delC, the allele frequency was 0.80% (20/2504), followed by 0.68% (17/2504) for c.109G>A, c.511_512insAACG for 0.12% (3/2504), c.299_300delAT for 0.04% (1/2504).

**Table 2:**
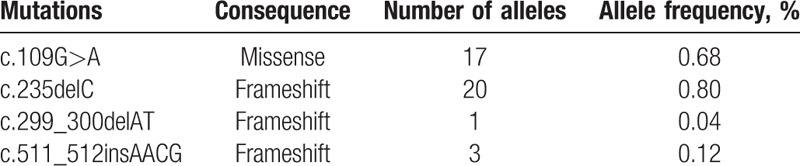
Allele frequencies of *GJB2* mutations in 1252 Hakka newborns.

Thirty-five newborns carried monoallelic variants in the heterozygous form: 19 with c.235delC, 12 with c.109G>A, 3 with c.511_512insAACG, and 1 with c.299_300delAT. Two newborns carried homozygous mutation for c.109G > A and 1 with c.235delC heterozygote compound c.109G > A heterozygote. Totally, 38 participants had molecular defects in *GJB2* gene (Table 3).

**Table 3:**
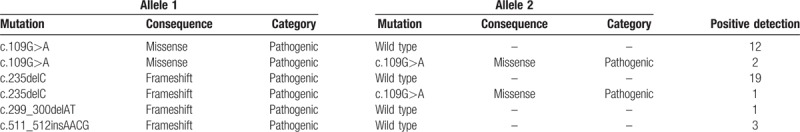
Identified *GJB2* genotypes in the studied 1252 Hakka Chinese newborns.

### 3.2 Mutations in *SLC26A4* gene

Eleven variants were identified in this cohort, including 5 missense mutations (c.589G > A, c.2168A > G, c.1229C > T, c.697G > C, c.1160C > T), 2 splice site mutations (c.IVS7-2A > G, c.IVS16-6G > A), 2 frameshift mutations (c.1975G > C, c.387delC), 1 nonsense mutation (c.2086C > T), and 1 variant in intron (c.919-18T > G). The mutant alleles of *SLC26A4* accounted for 1.84% (46/2504) of the total alleles in all subjects (Table 4). The most common mutation allele of *SLC26A4* in Hakka area was c.919-18T > G with a mutant frequency of 0.56% (14/2504). The second common mutation allele was c.IVS7-2A > G, the allele frequency was 0.52% (13/2504).

**Table 4:**
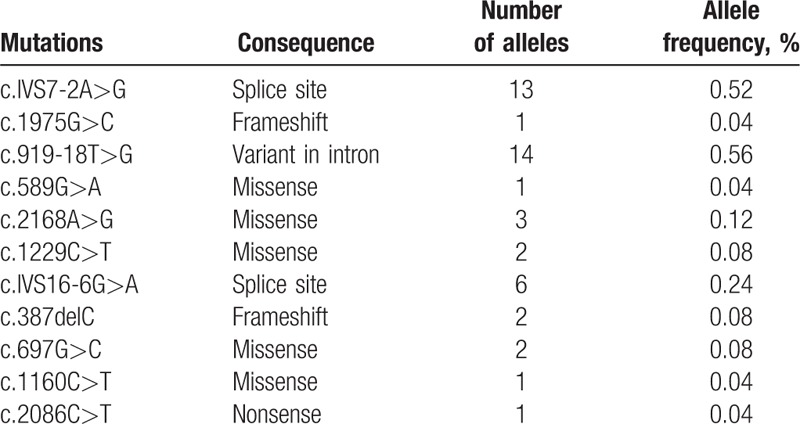
Allele frequencies of *SLC26A4* mutations in 1252 Hakka Chinese newborns.

Forty-two newborns carried monoallelic variants in the heterozygous form. One newborns carried homozygous mutation for c.IVS7-2A > G and 1 with c.2168A > G heterozygote compound c.1229C > T heterozygote. Totally, 44 subjects had molecular defects in *SLC26A4* gene (Table 5).

**Table 5:**
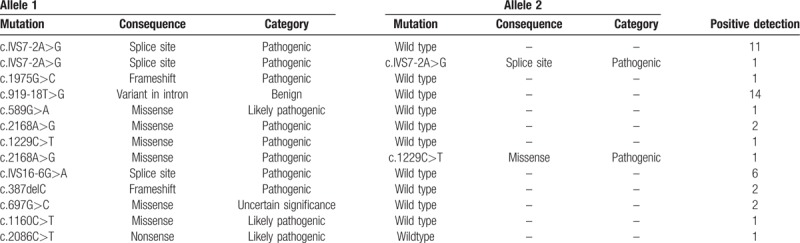
Identified *SLC26A4* genotypes in 1252 Hakka Chinese newborns.

### 3.3 Mutations in *GJB3* and mitochondrial genes

Two neonates carried mutations in *GJB3*, one was heterozygous mutation for c.538C > T and another one with c.547G > A heterozygote. In addition, 11 subjects were detected to be mitochondrial gene mutation carriers, accounting for 0.88% (11/1252) of the group. Eight newborns carried mutations in *MT-RNR1*, containing 1 heteroplasmic mutation for m.827A > G, 2 homoplasmic mutation for m.827A > G, 4 homoplasmic mutation for m.1555A > G, and 1 homoplasmic mutation for m.1494C > T. One newborns carried a heteroplasmic mutation for m.3243A > G in *MT-TL1.* Another 2 newborns carried a homoplasmic mutation for m.7444G > A in *MT-CO1.* Since the mutation of mitochondrial gene *MT-RNR1* is important mechanism of genetic susceptibility to aminoglycoside ototoxicity, screened carriers were provided with detailed drug using guide.

## 4 Discussion

Previous studies have reported that *GJB2*, *SLC26A4*, *GJB3*, and mitochondrial genes are the most common causes NSHL in Chinese people.^[14,29–31]^ Here we studied retrospectively 100 loci of 18 genes known to cause hearing impairment. The actual hearing tests of the subjects were not complete in our study. This is a limitation due to our study design.

### 4.1 *GJB2* mutation analysis

In the present study, *GJB2* mutations were detected in 3.04% (38/1252) of all subjects. Like most areas of China,^[8,32,33]^ the c.235delC was the most prevalent mutation in Hakka population with a carrier rate of 1.60% (20/1252), this result was similar with one study that c.235delC mutation carrier rate in the Chinese hearing population is 1.76% (198/7263).^[34]^ The second frequent mutation was c.109G > A, with a carrier rate of 1.20% (15/1252). We followed up 2 newborns with homozygous mutation in c.109G > A, the result was that they have normal hearing at the time of 9 months after birth. Another infant who with c.235delC heterozygote compound c.109G > A heterozygote also has normal hearing (Table 6). The mutation in c.109G > A is common in East Asians. The mutation frequency of c.109G > A in deaf population had been reported to be 4.2% in China,^[9]^ 4.3% in Thailand,^[35]^ 1.0% in Japan,^[36]^ and 0.6% in Korea.^[37]^ At present, the pathogenicity of this mutation site is controversial, because some individuals with normal hearing also carry the homozygous mutation.^[38,39]^

**Table 6:**
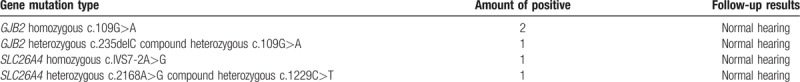
Hearing in 5 subjects with single-gene homozygous/compound heterozygous mutations from the 1252 Hakka Chinese newborns studied.

### 4.2 *SLC26A4* mutation analysis

In this study, *SLC26A4* with higher mutation rate 3.51% (44/1252) compared to *GJB2* 3.04% (38/1252). The mutation hotspots of *SLC26A4* differed among different nations and areas. In our group, the hotspot mutation of *SLC26A4* was c.919-18T > G, with a carrier rate of 1.12% (14/1252), but this variant has been proposed to as benign variation according to the reported data (http://deafnessvariationdatabase.org/). The carrier rate of c.IVS7-2A > G was 0.96% (12/1252), was similar with the study that c.IVS7-2A > G mutation carrier rate in the Chinese hearing population is 1.24% (90/7263).^[34]^ We followed up 1 subject with homozygous mutation in c.IVS7-2A > G; the result was that the infant has normal hearing at the time of 7 months after birth. Another infant with c.2168A > G heterozygote compound c.1229C > T heterozygote also has normal hearing (Table 6). Pendred syndrome (PDS) is classically described as bilateral sensorineural hearing loss and thyroid enlargement, and PDS is caused by mutations of *SLC26A4* gene, disease may occurs at any age from birth to adolescence, inducement including colds, fever, mild craniocerebral trauma, barotrauma, or other causes of increased intracranial pressure.^[40]^ We have informed the parents of the subject with homozygous mutation in c.IVS7-2A > G, to closely observe the child's behavior and its response to sound. If in doubt the parents should make a new hospital appointment, and we will follow-up this case next time.

### 4.3 *GJB3* and mitochondrial genes mutation analysis

In our study, 2 subjects were found carrying mutation in *GJB3*, one was heterozygous mutation for c.538C > T and another one was heterozygous mutation for c.547G > A. This result indicates that deafness-associated variation in *GJB3* was considered not common in Hakka population. The mutations of m.1555A > G and m.1494C > T for the *MT-RNR1* gene are considered as the most common mutations of mitochondrial genes. The mutation frequency of m.1555A > G was observed in 2.9% in China, Japan 3%,^[41]^ and Indonesia 5.3%.^[42]^ In our study, the mutation carrier rate of m.1555A > G and m.1494C > T accounted for 0.32% (4/1252) and 0.08% (1/1252), respectively, was similar with the study that m.1555A > G and m.1494C > T mutation carrier rate in the Chinese hearing population are 0.25% (18/7263), 0.04% (3/7263), respectively.^[34]^ Since the mutation of mitochondrial gene *MT-RNR1* is important mechanism of genetic susceptibility to aminoglycoside ototoxicity, screened carriers should be provided with detailed drug using guide. In our study, we have given out a warning to these carriers’ parents that these children may be prone to ototoxic effects of aminoglycoside, and informed the physician.

## 5 Conclusions

Considering that 1 to 3 newborns in every 1000 could be hearing impaired, screening for these mutations causing genetic hearing loss is relevant and may be applied if the gene mutation spectrum of the Hakka Chinese population is known. With the results of our study, the basis for neonatal screening is laid: of the 1252 participants, 95 subjects carried deafness-causing genes mutations with the carrier rate 7.59%. The mutation frequencies of *GJB2*, *SLC26A4*, *GJB3*, and mitochondrial genes were 3.04%, 3.51%, 0.16%, and 0.88%, respectively.

## Acknowledgments

The author would like to thank other colleagues who were not listed in the authorship of Clinical Core Laboratory and Center for Precision Medicine, Meizhou People's Hospital (Huangtang Hospital), Meizhou Hospital Affiliated to Sun Yat-sen University for their helpful comments on the manuscript.

## Author contributions

Pingsen Zhao conceived and designed the experiments; Pingsen Zhao and Liubing Lan recruited subjects and collected clinical data. Pingsen Zhao and Lifang Lin conducted the laboratory testing and prepared the manuscript.

**Conceptualization:** Pingsen Zhao.

**Data curation:** Pingsen Zhao, Lifang Lin, Liubing Lan.

**Formal analysis:** Pingsen Zhao.

**Funding acquisition:** Pingsen Zhao.

**Investigation:** Pingsen Zhao.

**Methodology:** Pingsen Zhao, Lifang Lin, Liubing Lan.

**Project administration:** Pingsen Zhao.

**Resources:** Pingsen Zhao, Liubing Lan.

**Software:** Pingsen Zhao, Lifang Lin.

**Supervision:** Pingsen Zhao.

**Validation:** Pingsen Zhao, Lifang Lin, Liubing Lan.

**Visualization:** Pingsen Zhao.

**Writing – original draft:** Pingsen Zhao, Lifang Lin.

**Writing – review and editing:** Pingsen Zhao.

## References

[1] Kral A O’Donoghue GM Profound deafness in childhood. N Engl J Med 2010;363:1438–50. 20925546 10.1056/NEJMra0911225

[2] Hilgert N Smith RJ Van Camp G Forty-six genes causing nonsyndromic hearing impairment: which ones should be analyzed in DNA diagnostics? Mutat Res 2009;681:189–96. 18804553 10.1016/j.mrrev.2008.08.002 PMC2847850

[3] Morton CC Nance WE Newborn hearing screening—a silent revolution. N Engl J Med 2006;354:2151–64. 16707752 10.1056/NEJMra050700

[4] Wang QJ Zhao YL Rao SQ Newborn hearing concurrent gene screening can improve care for hearing loss: a study on 14,913 Chinese newborns. Int J Pediatr Otorhinolaryngol 2011;75:535–42. 21329993 10.1016/j.ijporl.2011.01.016

[5] Guan MX Fischel-Ghodsian N Attardi G Nuclear background determines biochemical phenotype in the deafness-associated mitochondrial 12S rRNA mutation. Hum Mol Genet 2001;10:573–80. 11230176 10.1093/hmg/10.6.573

[6] Zhao H Li R Wang Q Maternally inherited aminoglycoside-induced and nonsyndromic deafness is associated with the novel C1494T mutation in the mitochondrial 12S rRNA gene in a large Chinese family. Am J Hum Genet 2004;74:139–52. 14681830 10.1086/381133 PMC1181901

[7] Dai P Yuan Y Huang D Molecular etiology of hearing impairment in Inner Mongolia: mutations in SLC26A4 gene and relevant phenotype analysis. J Transl Med 2008;6:74. 19040761 10.1186/1479-5876-6-74 PMC2630943

[8] Yuan Y You Y Huang D Comprehensive molecular etiology analysis of nonsyndromic hearing impairment from typical areas in China. J Transl Med 2009;7:79. 19744334 10.1186/1479-5876-7-79 PMC2754984

[9] Dai P Yu F Han B GJB2 mutation spectrum in 2,063 Chinese patients with nonsyndromic hearing impairment. J Transl Med 2009;7:26. 19366456 10.1186/1479-5876-7-26 PMC2679712

[10] Morton NE Genetic epidemiology of hearing impairment. Ann N Y Acad Sci 1991;630:16–31. 1952587 10.1111/j.1749-6632.1991.tb19572.x

[11] Smith RJ Bale JF Jr White KR Sensorineural hearing loss in children. Lancet 2005;365:879–90. 15752533 10.1016/S0140-6736(05)71047-3

[12] Rennels M Pickering LK Sensorineural hearing loss in children. Lancet 2005;365:2085–6. 10.1016/S0140-6736(05)66724-4 15964436

[13] Guo YF Liu XW Guan J GJB2, SLC26A4 and mitochondrial DNA A1555G mutations in prelingual deafness in Northern Chinese subjects. Acta Otolaryngol 2008;128:297–303. 18274916 10.1080/00016480701767382

[14] Dai P Li Q Huang D SLC26A4 c.919-2A>G varies among Chinese ethnic groups as a cause of hearing loss. Genet Med 2008;10:586–92. 18641518 10.1097/gim.0b013e31817d2ef1

[15] Yuan Y Guo W Tang J Molecular epidemiology and functional assessment of novel allelic variants of SLC26A4 in non-syndromic hearing loss patients with enlarged vestibular aqueduct in China. PLoS One 2012;7:e49984. 23185506 10.1371/journal.pone.0049984 PMC3503781

[16] Qu C Sun X Shi Y Microarray-based mutation detection of pediatric sporadic nonsyndromic hearing loss in China. Int J Pediatr Otorhinolaryngol 2012;76:235–9. 22154049 10.1016/j.ijporl.2011.11.009

[17] Hu X Liang F Zhao M Mutational analysis of the SLC26A4 gene in Chinese sporadic nonsyndromic hearing-impaired children. Int J Pediatr Otorhinolaryngol 2012;76:1474–80. 22796198 10.1016/j.ijporl.2012.06.027

[18] Xin F Yuan Y Deng X Genetic mutations in nonsyndromic deafness patients of Chinese minority and Han ethnicities in Yunnan, China. J Transl Med 2013;11:312. 24341454 10.1186/1479-5876-11-312 PMC3878508

[19] Dai P Yu F Han B The prevalence of the 235delC GJB2 mutation in a Chinese deaf population. Genet Med 2007;9:283–9. 17505205 10.1097/gim.0b013e31804d2371

[20] Liu X Dai P Huang DL Large-scale screening of mtDNA A1555G mutation in China and its significance in prevention of aminoglycoside antibiotic induced deafness [in Chinese]. Zhonghua Yi Xue Za Zhi 2006;86:1318–22. 16796900

[21] Jiang Y Huang S Deng T Mutation spectrum of common deafness-causing genes in patients with non-syndromic deafness in the Xiamen area, China. PLoS One 2015;10:e0135088. 26252218 10.1371/journal.pone.0135088 PMC4529078

[22] Wei Q Wang S Yao J Genetic mutations of GJB2 and mitochondrial 12S rRNA in nonsyndromic hearing loss in Jiangsu Province of China. J Transl Med 2013;11:163. 23826813 10.1186/1479-5876-11-163 PMC3706284

[23] Ma Y Xiao Y Bai X GJB2, SLC26A4, and mitochondrial DNA12S rRNA hot-spots in 156 subjects with non-syndromic hearing loss in Tengzhou, China. Acta Otolaryngol 2016;136:800–5. 27066914 10.3109/00016489.2016.1164893

[24] Rehman AU Bird JE Faridi R Mutational spectrum of MYO15A and the molecular mechanisms of DFNB3 human deafness. Hum Mut 2016;37:991–1003. 27375115 10.1002/humu.23042 PMC5021573

[25] Chen J Zheng H Bei JX Genetic structure of the Han Chinese population revealed by genome-wide SNP variation. Am J Hum Genet 2009;85:775–85. 19944401 10.1016/j.ajhg.2009.10.016 PMC2790583

[26] Wang WZ Wang CY Cheng YT Tracing the origins of Hakka and Chaoshanese by mitochondrial DNA analysis. Am J Phys Anthropol 2010;141:124–30. 19591216 10.1002/ajpa.21124

[27] Yuan Y Zhang X Huang S Common molecular etiologies are rare in nonsyndromic Tibetan Chinese patients with hearing impairment. PLoS One 2012;7:e30720. 22389666 10.1371/journal.pone.0030720 PMC3289614

[28] Yu F Han DY Dai P Mutation of GJB2 gene in nonsyndromic hearing impairment patients: analysis of 1190 cases [in Chinese]. Zhonghua Yi Xue Za Zhi 2007;87:2814–9. 18167282

[29] Mena R Aye M Genetics of human hereditary hearing impairment. Aye Med Coll Abbottabad 2017;29:671–6. 29331002

[30] Duan SH Zhu YM Wang YL Common molecular etiology of nonsyndromic hearing loss in 484 patients of 3 ethnicities in northwest China. Acta Otolaryngol 2015;135:586–91. 25761933 10.3109/00016489.2015.1006334

[31] Fang Y Gu M Wang C GJB2 as well as SLC26A4 gene mutations are prominent causes for congenital deafness. Cell Biochem Biophys 2015;73:41–4. 25649612 10.1007/s12013-015-0562-3

[32] Shen DL Wang B Bai J Clinical value of CYP2C19 genetic testing for guiding the antiplatelet therapy in a Chinese population. J Cardiovasc Pharmacol 2016;67:232–6. 26727381 10.1097/FJC.0000000000000337

[33] Chen K Zong L Liu M Developing regional genetic counseling for southern Chinese with nonsyndromic hearing impairment: a unique mutational spectrum. J Transl Med 2014;12:64. 24612839 10.1186/1479-5876-12-64 PMC3975227

[34] Yin A Liu C Zhang Y The carrier rate and mutation spectrum of genes associated with hearing loss in South China hearing female population of childbearing age. BMC Med Genet 2013;14:57. 23718755 10.1186/1471-2350-14-57 PMC3680026

[35] Wattanasirichaigoon D Limwongse C Jariengprasert C High prevalence of V37I genetic variant in the connexin-26 (GJB2) gene among non-syndromic hearing-impaired and control Thai individuals. Clin Genet 2004;66:452–60. 15479191 10.1111/j.1399-0004.2004.00325.x

[36] Abe S Usami S Shinkawa H Prevalent connexin 26 gene (GJB2) mutations in Japanese. J Med Genet 2000;37:41–3. 10633133 10.1136/jmg.37.1.41 PMC1734448

[37] Han SH Park HJ Kang EJ Carrier frequency of GJB2 (connexin-26) mutations causing inherited deafness in the Korean population. J Hum Genet 2008;53:1022–8. 19043807 10.1007/s10038-008-0342-7

[38] Pollak A Skorka A Mueller-Malesinska M M34T and V37I mutations in GJB2 associated hearing impairment: evidence for pathogenicity and reduced penetrance. Am J Med Genet A 2007;143A:2534–43. 17935238 10.1002/ajmg.a.31982

[39] Gallant E Francey L Tsai EA Homozygosity for the V37I GJB2 mutation in fifteen probands with mild to moderate sensorineural hearing impairment: further confirmation of pathogenicity and haplotype analysis in Asian populations. Am J Med Genet A 2013;161A:2148–57. 23873582 10.1002/ajmg.a.36042 PMC3745519

[40] Jacobs HT Hutchin TP Kappi T Mitochondrial DNA mutations in patients with postlingual, nonsyndromic hearing impairment. Eur J Hum Genet 2005;13:26–33. 15292920 10.1038/sj.ejhg.5201250

[41] Usami S Abe S Akita J Prevalence of mitochondrial gene mutations among hearing impaired patients. J Med Genet 2000;37:38–40. 10633132 10.1136/jmg.37.1.38 PMC1734443

[42] Malik SG Pieter N Sudoyo H Prevalence of the mitochondrial DNA A1555G mutation in sensorineural deafness patients in island Southeast Asia. J Hum Genet 2003;48:480–3. 12955586 10.1007/s10038-003-0056-9

